# Analysis and Simulation of the Compressive Strength of Bioinspired Lightweight Structures Manufactured by a Stereolithography 3D Printer

**DOI:** 10.3390/biomimetics9040240

**Published:** 2024-04-17

**Authors:** Cristina Alía García, Álvaro Rodríguez Ortiz, José Manuel Arenas Reina, Juan David Cano-Moreno, Manuel Gómez Gómez

**Affiliations:** Escuela Técnica Superior de Ingeniería y Diseño Industrial, Universidad Politécnica de Madrid, 28012 Madrid, Spain; cristina.alia@upm.es (C.A.G.); alvaro.rodriguez@upm.es (Á.R.O.); juandavid.cano@upm.es (J.D.C.-M.); manuel.gomezg@alumnos.upm.es (M.G.G.)

**Keywords:** metamaterial, bioinspiration, SLA 3D printer, finite element analysis, compressive strength, manufacturing costs

## Abstract

The use of metamaterials is a good alternative when looking for structures that can withstand compression forces without increasing their weight. In this sense, using nature as a reference can be an appropriate option to design this type of material. Therefore, in this work, a comparative study of a selection of eight representative models of a wide variety of existing solutions, both bioinspired and proposed by various researchers, is presented. These models have been manufactured using stereolithography (SLA) printing, which allows complex geometries to be obtained in a simple way that would be more complicated to achieve by other procedures. Additionally, the manufacturing cost of each model has been determined. The compression tests of the different models have made it possible to evaluate the breaking force and its corresponding deformation. Likewise, a finite element analysis of the manufactured models has been carried out to simulate their behavior under compression, achieving results very similar to those obtained in the experimental tests. In this way, it has been concluded that, among the three-dimensional patterns, the structure called “3D auxetic” is the one that supports the greatest breaking force due to the topographic characteristics of its bar structure. Similarly, among the two-dimensional patterns, the structure called “Auxetic 1”, with a topography based on curves, is capable of supporting the greatest deformation in the compression direction before breaking. Moreover, the highest resistance-force-to-cost ratio has been obtained with a “3D auxetic” structure.

## 1. Introduction

In the current context, protection against compressive forces has become a key area of study and development in various fields, such as civil engineering, aerospace, and the automotive industry, among others. The goal is to safeguard the lives and integrity of individuals, prevent damage to infrastructures, and minimize the consequences of these forces [1,2].

Furthermore, technological advancements and the constant pursuit of innovative solutions have led to the creation and discovery of new materials with extraordinary properties. Among these materials are metamaterials. Mechanical metamaterials are artificially designed and architected structures that derive their mechanical performance from the unique design of their unit cells [3]. These materials are designed to exhibit characteristics that conventional materials cannot. Due to their versatile behavior, metamaterials are divided into various main groups, including positive Poisson’s ratio (PPR) structures [4], zero Poisson’s ratio (ZPR) structures [5,6,7,8], and negative mechanical metamaterial indices such as auxetics [9,10,11], negative stiffness, and thermal expansion structures [12,13,14,15]. As a result, some of the features they can possess include ultra-high specific strength and a high volumetric modulus-to-shear modulus ratio, among others. These properties make metamaterials an ideal choice for creating structures that absorb and attenuate the effects of compressive forces [16,17,18]. Hence, they are employed in various applications such as sports equipment [19], damping vibration [20], actuators [21] or sound absorption [22,23], providing good compressive strength while maintaining a low weight.

On the other hand, observing and imitating nature is another method for scientists and engineers to find solutions to problems. Animals, plants and microorganisms have evolved over millions of years, developing efficient mechanisms to survive environmental challenges. The strategies organisms use to resist and absorb compressive forces can serve as inspiration for improving or developing new structures. For example, bamboo vascular bundles have been idealized to create honeycomb tubular structures to enhance the energy absorption capacity of vehicle bumpers [24]. The luffa plant inspired the creation of nickel-plated luffa sponge structures with higher energy absorption capacity and toughness compared to other engineering materials like aluminum foam and metal lattice materials [25]. Butterfly wings, with their excellent bending properties, have also been reproduced for engineering applications [26].

In addition, a fundamental tool for development in various scientific and technological fields is finite element analysis (FEA). By using calculations, models, and simulations, FEA predicts the behavior of an object under different physical conditions. Its use can reduce the number of physical prototypes created and experiments conducted, evaluate alternative materials, optimize designs, analyze different solutions quickly and decrease the time and costs associated with design and manufacturing [27].

The advent of 3D printing has revolutionized the manufacturing field, enabling the direct and efficient creation of prototypes and products. This technology allows for the design and manufacture of complex geometries and lightweight structures with high strength, which are challenging to produce using conventional machining and processing techniques [28]. It also enables cost-effective mass customization and reduces the amount of material used during production, as the material is applied only where needed [29,30,31]. In this regard, the use of 3D printing to manufacture metamaterials is widely documented in the literature. Li et al. [32] use SLS technology to fabricate beam lattice structures (BLS) with a negative Poisson’s ratio (NPR) effect by applying sinusoid-shaped beams, conducting experimental compression tests and subsequently validating with finite element analysis. Alomarah et al. [33] employ Multi-Jet Fusion (MJF) to manufacture auxetic structures for compression tests. Hamzehei et al. [34] present new 3D metamaterials with zero Poisson’s ratio for energy absorption applications using SLA 3D printing. Sahin et al. [35] use a new 4D-printing method by rotating the printed axis of bilayers inspired by the large-flowered butterwort (Pinguicula grandiflora). However, when using this type of 3D printing, the associated manufacturing costs are often not considered despite their importance as another variable to consider.

Therefore, the objective of this article is to carry out a technical-economic analysis of bioinspired light structures manufactured by an SLA 3D printer that integrates the results of the compression tests of each support with the evaluation of the cost associated with its manufacture to facilitate the choice of the alternative that best combines the “mechanical resistance force to cost” relationship.

As the number of bioinspired light structures that can be considered is very large, in the present work, eight representative structures have been selected that present a wide functional and formal diversity (five with a two-dimensional pattern and three with a three-dimensional pattern). For each of these structures, their manufacturing cost has been calculated and their mechanical resistance to compression has been determined both experimentally and through finite element simulation using the ABAQUS software (2023 version) [36].

In the selection of the eight representative models, efforts have been made to ensure that there is functional and formal diversity of structures, both with two-dimensional and three-dimensional patterns, that are adequate to resist compression forces. Additionally, the selected models are especially appropriate for manufacturing using 3D printing by stereolithography (SLA).

In this way, for two-dimensional patterns, a first conventional rhombus structure is proposed (model A) that allows behavior with a certain elasticity. Along these lines, the hexagonal structure (model B) presents a somewhat more complex geometry, although with better mechanical behavior. The selection of auxetic geometries allows considering non-conventional structures with a specific mechanical behavior (auxetic models are compressed in their horizontal direction when subjected to a vertical compression stress). In this way, two representative models of auxetic structures have been chosen: models C (cells with smooth curves) and D (stackable cells). Additionally, in order to evaluate whether there is an influence of changes in direction on the propagation of the breakage on the failure stress, a structure based on the Voronoi geometry (model E) characterized by its high degree of irregularity has been selected.

On the other hand, in the case of three-dimensional patterns, a model with a conventional cubic structure and diagonal reinforcements (F models), a three-dimensional auxetic model (G model) and a bioinspired model with a very irregular structure and unpredictable mechanical behavior (model H).

## 2. Materials and Methods

### 2.1. Manufacturing of Bioinspired Models

**Figure 1:**
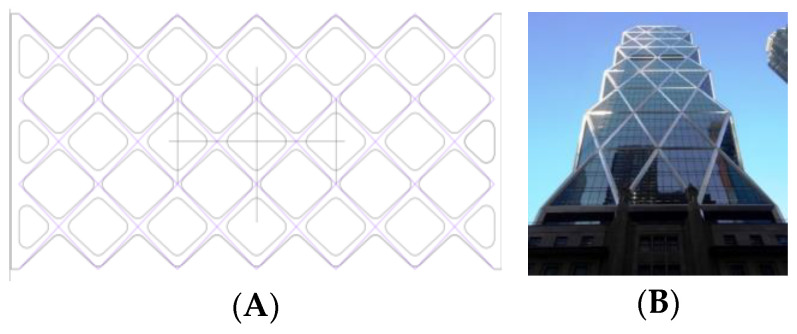
Rhombuses model (**A**) and Hearst Tower (**B**).

**Figure 2:**
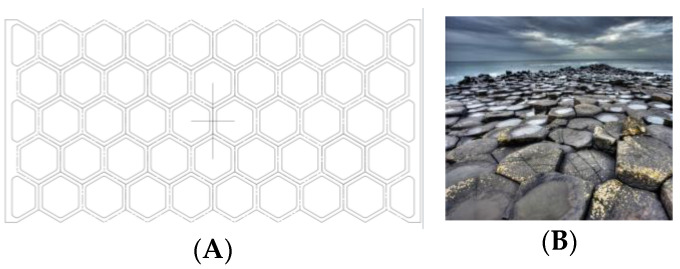
Hexagonal model (**A**) and basalt formations (**B**).

**Figure 3:**
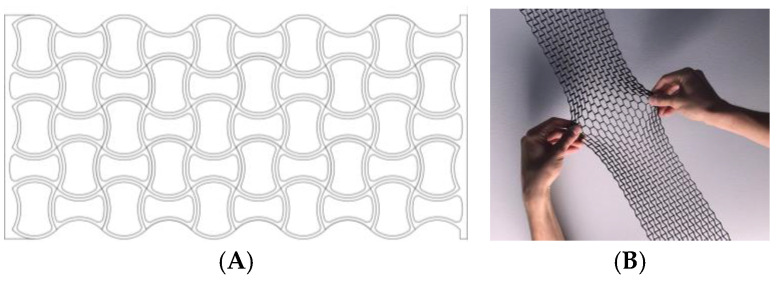
Auxetic 1 model (**A**) and auxetic pattern study (**B**).

**Figure 4:**
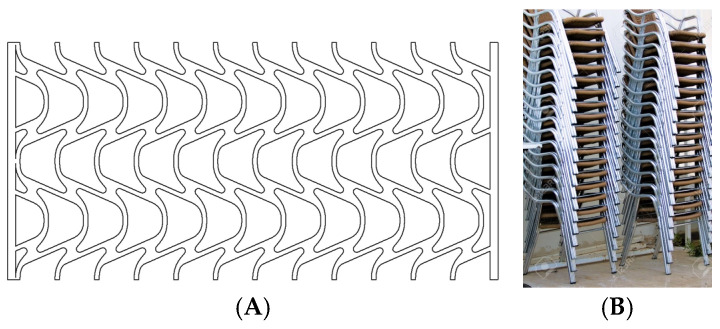
Auxetic 2 model (**A**) and stacked chair system (**B**).

**Figure 5:**
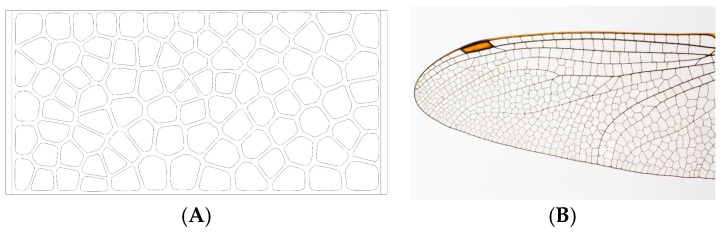
Organic Voronoi model (**A**) and insect wing (**B**).

**Figure 6:**
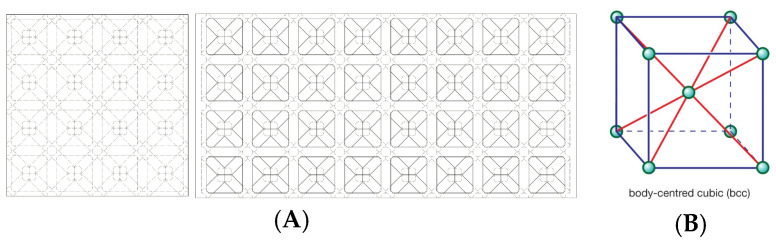
Cubic with diagonals model (**A**) and BCC structure (**B**).

**Figure 7:**
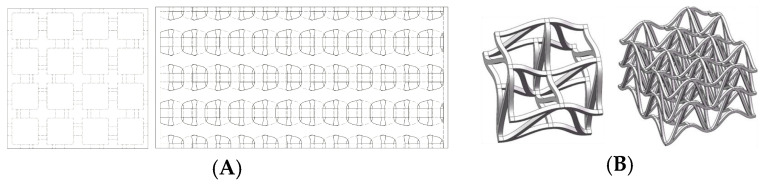
Three-dimensional auxetic model (**A**) and mechanical properties of 3D double-U structures (**B**) [39].

**Figure 8:**
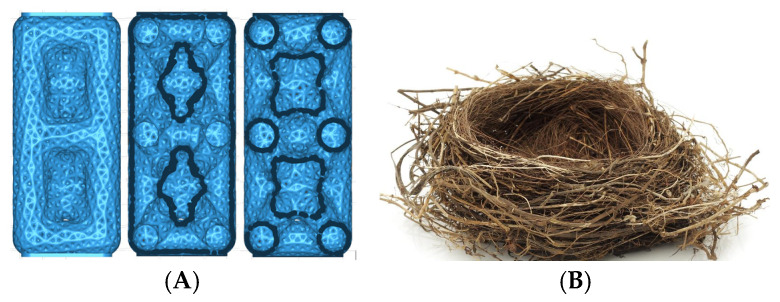
Organic model with irregular mesh (**A**) and bird nest (**B**).

The criterion followed for the design of the structures has been the combination of existing models in the literature (models A, C, D, F, G [37,38,39]) and, in some cases, bio-inspiration (models B, E, and H). Thus, 8 different models have been created, aiming to represent the functional and formal diversity that structural elements subjected to compression forces may exhibit. Furthermore, the various designed models have been grouped into two patterns. On one hand, the structures with a two-dimensional pattern (2D) developed by repeating the unit cells along with the two principal in-plane directions and then extruding in a third direction [40]. On the other hand, the structures with a three-dimensional pattern (3D) are designed by repeating the unit cells along with the three principal directions [41]. The following is an explanation of each of the 8 models divided into 2D and 3D patterns:

1. Two-Dimensional Patterns:
  - Rhombuses. It presents a grid with ribs in two directions perpendicular to each other and rounded corners (Figure 1). This model has a relatively conventional geometry that may be reminiscent of mobile accordion-type structures such as folding fences or architectural elements such as the facade of the Hearst Tower.
  - Hexagonal. It presents a grid similar to the structure of honeycombs or basalt formations (Figure 2). Although the advantages of this type of hexagonal structure are successfully applied to honeycomb boards, in this study, they are subjected to stress in their most unfavorable direction.
  - Auxetic 1. This geometry was chosen following an auxetic pattern typical of literature due to its simplicity, regularity and sense of continuity given by the curved shapes. Said model will be compressed in its horizontal direction when subjected to a vertical compression stress (Figure 3).
  - Auxetic 2. Given the variety of authentic patterns that exist, a second representative model of this type of metamaterials has been selected (Figure 4). The chosen geometry is inspired by stacking systems such as glasses or chairs.
  - Organic Voronoi. This model follows a Voronoi geometry and is characterized by irregularity (Figure 5). The chosen geometry is inspired by the fur of giraffes or the wings of some insects.
2. Three-Dimensional Patterns:
  f. Cubic with diagonals. This model follows a conventional geometry following the repetition of cells formed by cylindrical bars in a cubic arrangement with reinforcements on their diagonals (Figure 6). The chosen geometry is inspired by the BCC crystalline atomic structure.
  g. Three-dimensional (3D) auxetic. For this model, a design from the literature [42] has been adapted, modifying the bar-shaped structure to a structure with more continuity that gives the sensation of being made up of two-dimensional elements. In this proposal, axial compression generates compression in the other two directions of space (Figure 7), while the model (auxetic 2) only compresses one direction indirectly.
  h. Organic (irregular mesh). To design this model, the base pattern created for model F, which is slightly elongated in the vertical direction, was used as a starting point. Said structure would be figuratively wrapped in an organic and irregular mesh, inspired by how a spider wraps prey that falls into its web or bird nests (Figure 8).

**Figure 9:**
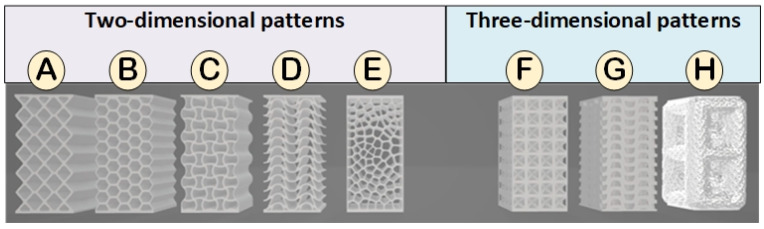
Designed models: (**A**) rhombuses, (**B**) hexagonal, (**C**) auxetic 1, (**D**) auxetic 2, (**E**) organic Voronoi, (**F**) cubic with diagonals, (**G**) three-dimensional auxetic and (**H**) organic with irregular mesh.

Once the models are selected by combining literature and bioinspiration, the 8 designs are modeled through different parametric design tools: Auto-desk Inventor, Autodesk Fusion 360, Autodesk Meshmixer and Chitubox. Figure 9 shows the modeling result of each of the designs. Furthermore, in order to compare the models with each other, all structures share maximum dimensions of 50 × 50 × 102.67 [mm] and a net volume of 77.88 mL ± 1%.

### 2.2. Equipment

**Figure 10:**
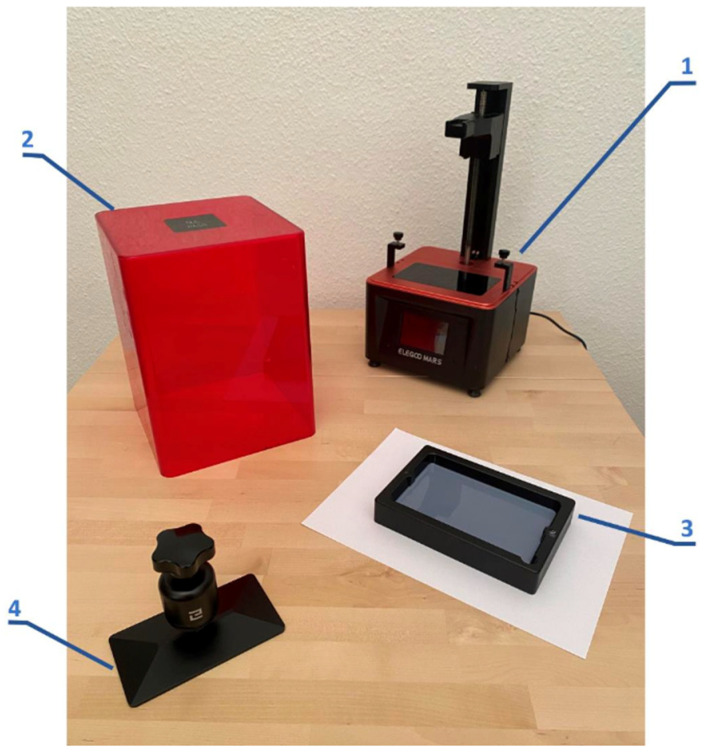
Main components of the printer: (1) main body, (2) protective cover with UV filter, (3) bucket and (4) manufacturing platform.

Once the bioinspired structures are selected, they are manufactured using a first-generation stereolithography (SLA) 3D printer (ELEGOO, Shenzhen, China), which uses a 4.72 × 2.68 square inch LCD screen and a workspace height of 6.1 inches. Figure 10 shows the main components of the SLA printer.

The printing parameters used to manufacture the different structures have been kept the same for all designs in order to allow for a comparison of all models. Table 1 shows the printing parameters.

For the tests, a model TN-MD machine (HOYTOM, S.L., Bilbao, Spain), motorized with automatic control via a computer, was used. Its capacity is 200 kN, the piston stroke length is 125 mm, and the displacement rate was fixed at 2 mm/min. Three tests have been carried out on each of the designed models.

### 2.3. Materials

The material used was a standard photopolymer resin (ELEGOO, Shenzhen, China) for SLA printing [43]. This resin has high fluidity and low viscosity, fast photocuring ability, high accuracy printing, good prototyping effect, easy cleaning and curing and wide applications. Once the specimen is printed it must be washed using an isopropyl alcohol solution for a short period of time, as described by the provider. This resin is commonly used to prototype parts that would be made from polypropylene (PP). The use of this resin is due to the fact that its mechanical properties [44] seem to be suitable for use in elements that will be subjected to large deformations or impacts, which is the potential application. Table 2 shows the technical characteristics of the resin used.

Table 3 specifies, for each of the eight structure models manufactured, the main dimensions of each lattice, the diameter of the reinforcement, the mass and the density.

### 2.4. Manufacturing Costs

The determination of the manufacturing cost in 3D printing manufacturing processes is a relevant element in evaluating the technical-economic viability of the parts obtained by these procedures [45]. In this way, the model that best combines mechanical performance and adaptation to the SLA manufacturing process can be selected.

The manufacturing cost has been divided into the cost of the material used in the model, cost of labor and cost of operating the workplace. The SLA printer software (Elegoo Mar 5 version) itself allows you to determine the mass of resin used and the printing time. This software also provides the material cost (including the material used as a support, which, in this case, is the same resin) when the type of resin and the cost per unit of mass (EUR/kg) are indicated. The cost of labor is calculated as a product of the printing time times the weighted salary of the operator for this type of process. The weighted day considers that the operator only dedicates a small fraction of the model processing time to attending to the 3D printer (mainly for starting up the machine, periodic supervision and performing post-curing), dedicating the rest of the time to other processes and activities. In this way, the weighted salary is obtained as a product of the standard salary for the aforementioned fraction of time dedicated to the model development process (in our case, this weighted salary is 5.30 EUR/hour). The most relevant factors of the operating cost of the 3D printer are the electrical energy consumption and the amortization of the machine. Considering the energy consumed per hour of the machine and that the expected amortization period is four years, the operating cost per hour of the machine has been estimated at 0.035 EUR/hour. The operating cost for processing each model is obtained as a product of the operating cost/hour times the corresponding printing time. Table 4 shows the detailed manufacturing costs (material, labor and operating costs) for each of the eight models manufactured by SLA.

### 2.5. Methodology of Finite Element Analysis (FEA)

**Figure 11:**
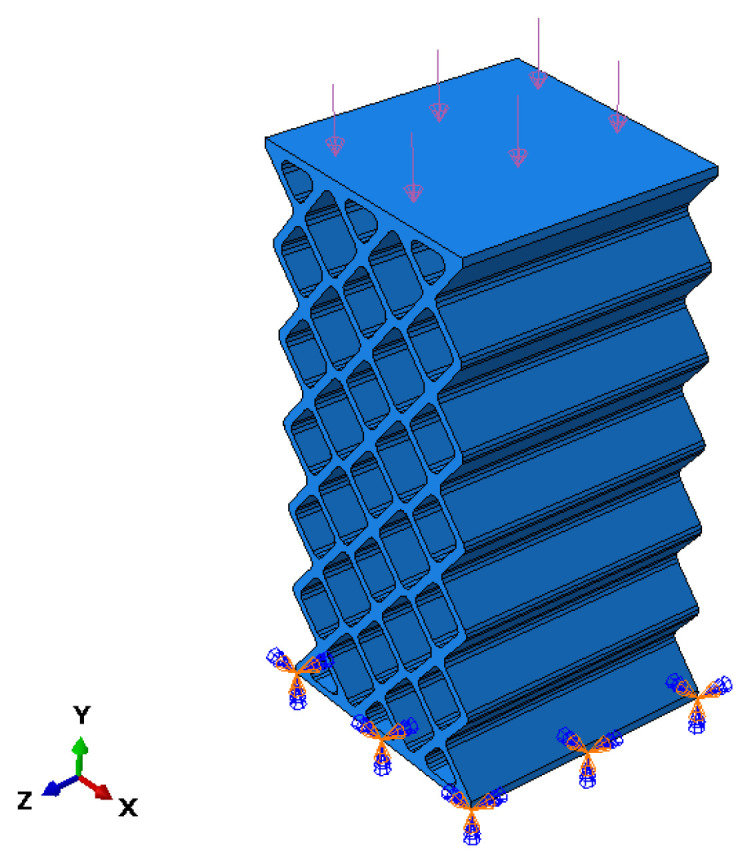
Boundary conditions in the FEM model A (rhombuses).

The finite element analysis of the most representative models (A, B, C, D, E, F, G) has been carried out. To do this, the experimental compression test was considered, where the specimen was supported on its lower base while the piston of the testing machine came into contact with a downward movement on its upper face. To replicate these boundary conditions in the FEM model, an embedment condition has been placed on the lower base, and pressure has been applied to the upper face (Figure 11).

The pressure value that has been considered for each model is the equivalent of the average failure force recorded in the experimental tests.

**Figure 12:**
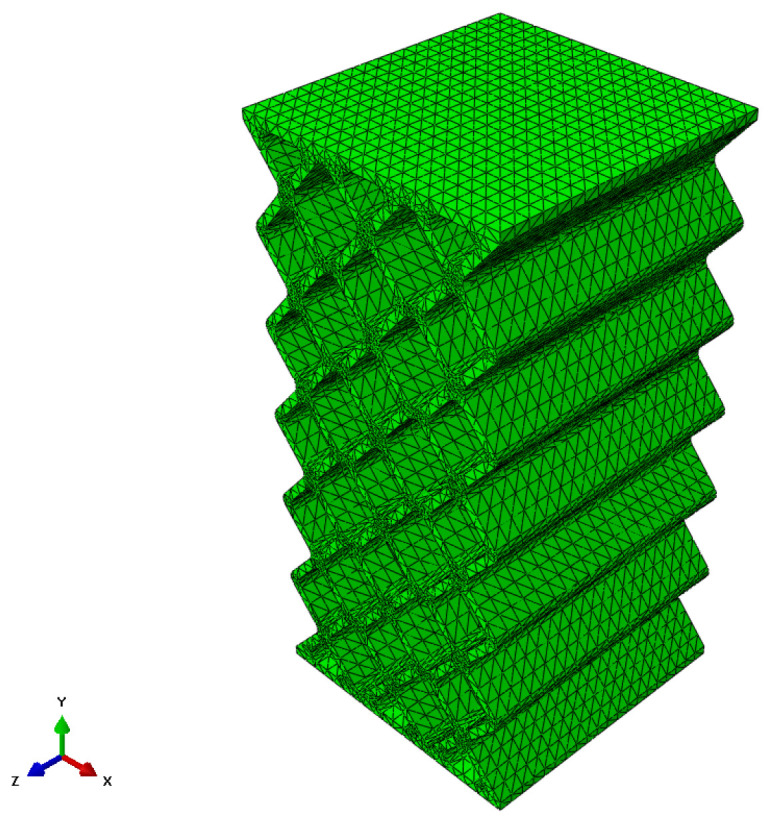
Mesh in the FEM model A (rhombuses).

The mesh used is the result of a compromise between the available computing power and the maximum quality of the mesh. The quality of the mesh is determined by the size, distribution and type. In terms of size, we chose one that was equivalent to the minimum thickness that existed in the model. The distribution of the elements can be structured or free. ABAQUS allows a structured distribution when the models have regular shapes. In this case, this technique was available for some models, so for the analysis, it was decided to use the free distribution in all models. When using a free distribution, the elements have to be tetrahedral, leaving it to be decided whether they have to be linear or quadratic. The difference between the two is the number of existing nodes per element, being 4 for the linear ones and 10 for the quadratic ones. The decision was made to use quadratic elements, which, although they require more calculation resources, result in greater precision. In Figure 12, you can see the result of the meshing for model A, where C3D10-type elements have been used.

## 3. Results

### 3.1. Experiment Results

Once the tests carried out and the models used have been described, this section will show both the results obtained and their analysis. The different models used will allow us to study how each of the patterns behaves when subjected to compression stresses.

**Figure 13:**
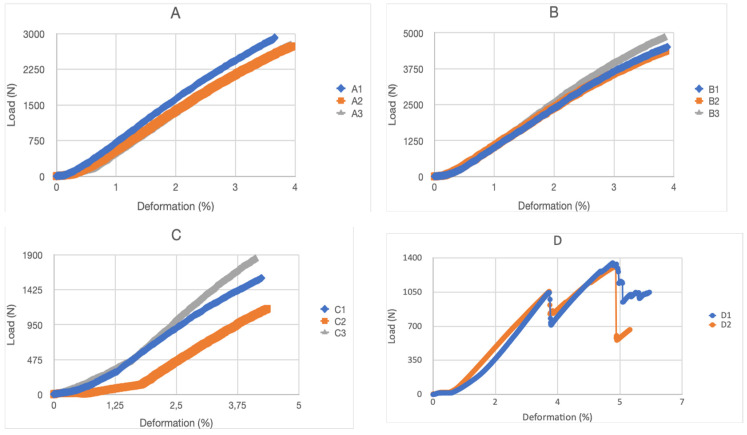

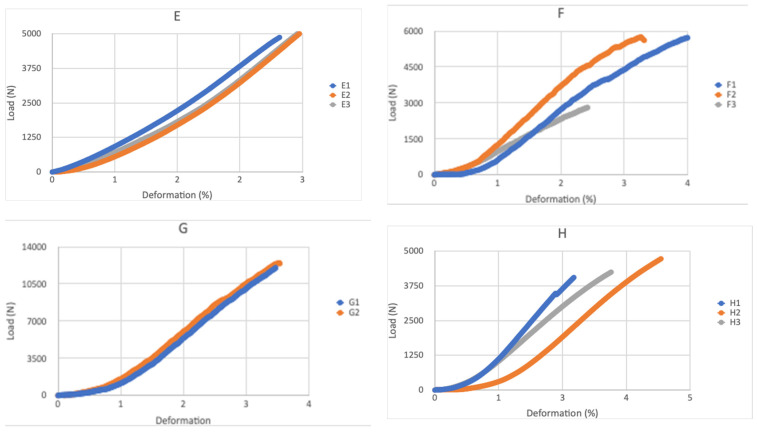
Force–displacement curves for proposed structure models: (**A**) rhombuses, (**B**) hexagonal, (**C**) auxetic 1, (**D**) auxetic 2, (**E**) organic Voronoi, (**F**) cubic with diagonals, (**G**) three-dimensional auxetic and (**H**) organic with irregular mesh.

Figure 13 shows the force–displacement curves of the specimens tested in compression. It can be seen that in all cases, except in model D (auxetic 2), the force increases to a maximum value where breakage occurs. In the case of model D, due to its layer configuration, it makes the breakage different, having an intermediate partial breakage before the total breakage. Furthermore, differences in the maximum supported force are observed between the different models and an increase in rigidity is observed as a function of deformation. This aspect is apparently linked to local geometric nonlinearity effects and is very different from that usually found in compact materials. Thus, in structures in which the applied loads produce deformations of great magnitude, such that the hypothesis that the final deformed position coincides with the initial position cannot be accepted. This fact makes the response of the structure highly nonlinear, and it is common to call this phenomenon geometric nonlinearity [46]. Furthermore, this nonlinearity affects the mode of failure that occurs and the speed at which it occurs, causing total failure, as shown in the graphs.

After carrying out the mechanical compression tests, the experimental results shown in Table 5 have been obtained.

**Figure 14:**
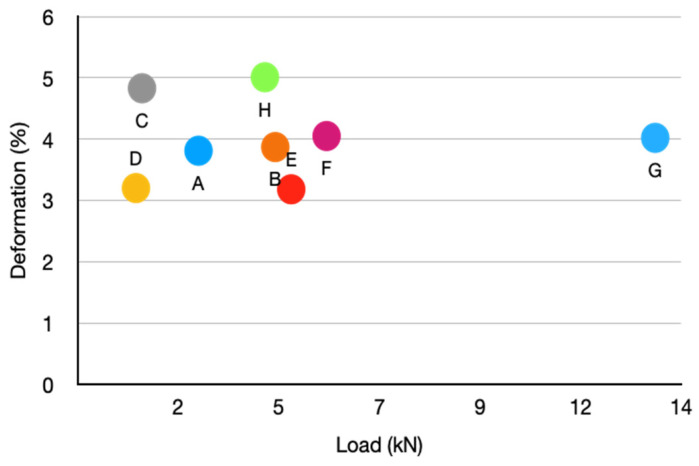
Comparative diagram between breaking force and breaking deformation for the eight models considered (A to G).

Figure 14 shows a correlation diagram that relates the failure stress (in %) to the compression failure load for the eight models considered. In the case of two-dimensional patterns, which offer anisotropic properties intended for unidirectional loads, it is observed that model E (organic Voronoi) has the highest failure load while the auxetic models (D and C) have the lowest load they support. Regarding the breaking deformation, all models with a two-dimensional pattern present a deformation between 3.18% and 4.83%.

In the case of three-dimensional patterns intended to support 3D loads, it is observed that model G (3D Auxetic) stands out for its high breaking load (13.39 kN), while model H (organic and irreg. mesh) is the one that presents the least load. The fracture stress values for the three-dimensional pattern models are between 4.02% and 5.01%.

An analysis of the breaking force and the manufacturing cost of each of the models has also been conducted (Table 6). In this way, it is observed that model G (Three-dimensional auxetic) is the one with the highest manufacturing cost. The rest of the models, except for the H model (organic with irregular mesh), whose cost is also high, have a similar cost, although there is a difference in the breaking forces that they are capable of withstanding. This comparison is very practical when the economic part is relevant in decision-making. If we compare the force/cost ratio, model G presents the best value, eight times higher than the D model and five times higher than the C model.

**Figure 15:**
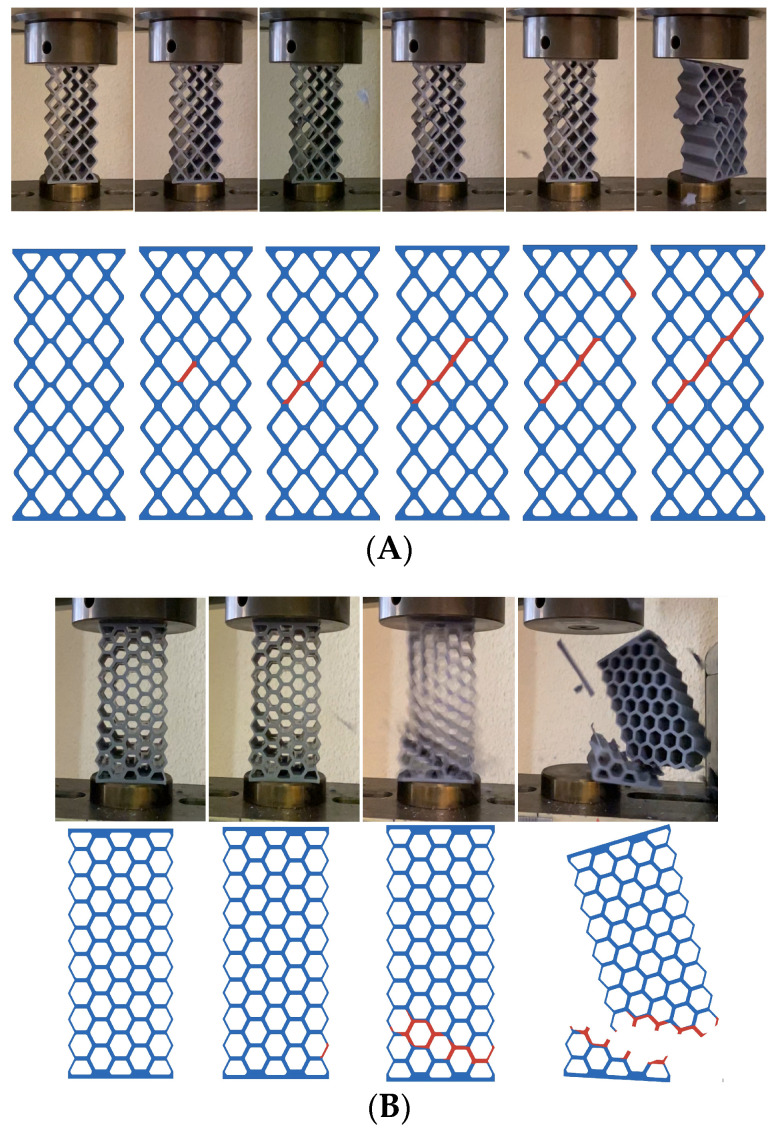
Breakage type of designs (**A**) (rhombuses) and (**B**) (hexagonal).

On the other hand, the type of breakage observed in each model is analyzed. The fracture line followed by each model is shown in red. In the case of models A (rhombuses) and B (hexagonal), the angle of failure is 45°, as are materials with ductile behavior (Figure 15).

**Figure 16:**
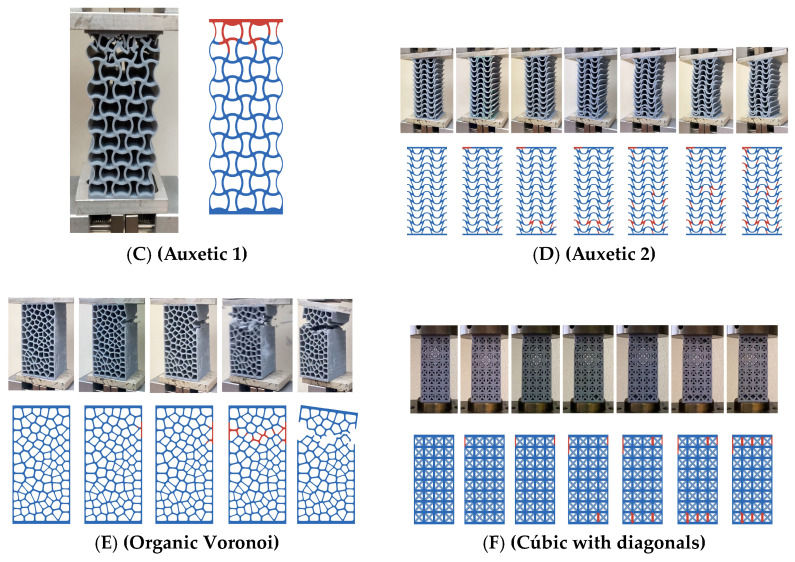
Breakage type of designs: (**C**) auxetic 1, (**D**) auxetic 2, (**E**) organic Voronoi and (**F**) cubic with diagonals.

However, models C (auxetic 1), D (auxetic 2), E (organic Voronoi) and F (cubic with diagonals) have a fracture angle of 90° typical of brittle materials (Figure 16).

**Figure 17:**
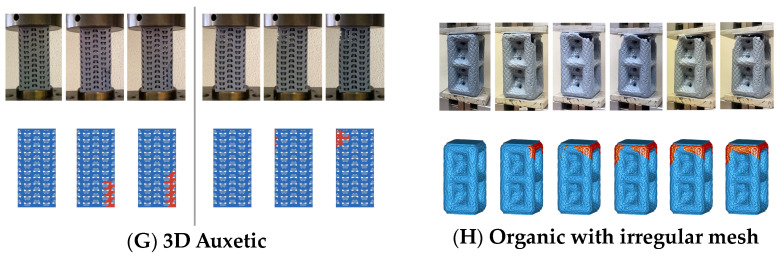
Breakage type of designs: (**G**) 3D auxetic and (**H**) organic with irregular mesh.

On the other hand, in models G (three-dimensional auxetic) and H (organic with irregular mesh), the breakage begins in one corner of the model, and then the propagation begins (Figure 17).

Table 7 shows a summary of the breakage types obtained. Therefore, there are very different fracture behaviors depending on the model design.

### 3.2. Results of Finite Element Analysis (FEA)

This section describes the results obtained when applying finite element analysis (FEA) to the structural models considered in Section 2.5.

**Figure 18:**
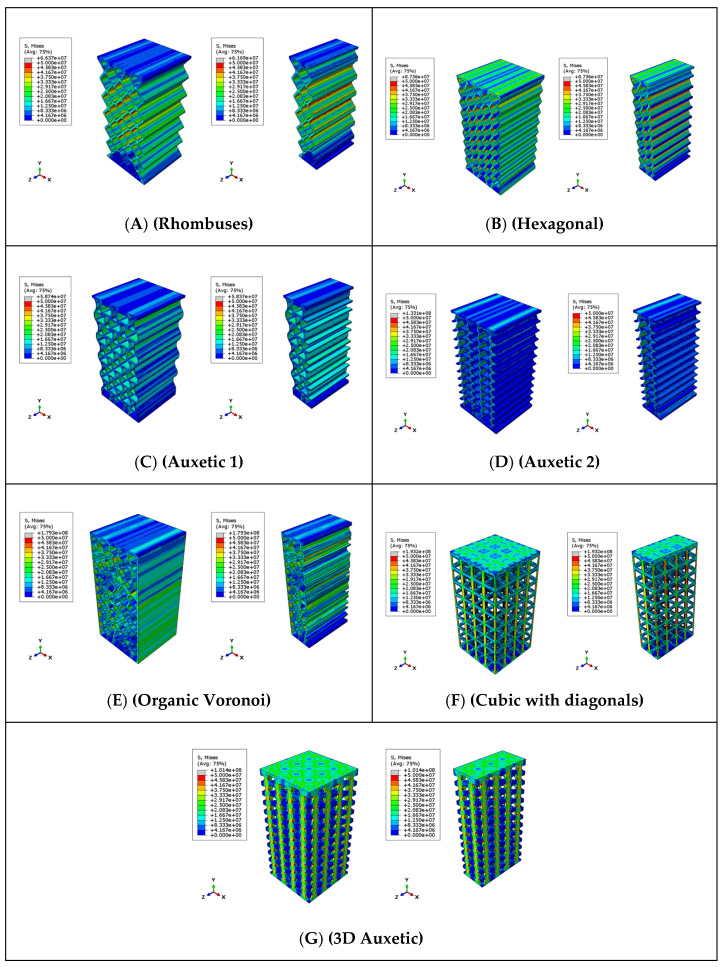
Stress field of finite element models simulated with ABAQUS.

With these conditions, the results of the finite element analysis are shown. The stress field corresponding to the Von Misses stresses (units in Pascals) is shown for both the complete specimen and the middle section for each of the analyzed models (Figure 18). According to the manufacturer, in its technical sheet, the compressive strength is between 36 and 53 MPa, and the bending strength is between 59 and 70 MPa. The stress state of the test specimen is a mix of compression and traction stress states depending on the areas. The failure criterion is an average value of the minimum and maximum failure stresses provided by the manufacturer ((70 + 36)/2 = 53 ~ 50 MPa). With this criterion, the test specimen collapses when any area reaches 50 MPa von Misses stresses. The visualization of the stresses in the ABAQUS models was set, placing the visualization between 0 MPa (blue color) and 50 MPa (red color).

The models have been loaded by using a linear increase in force. Figure 18 shows the moment in which the areas that reach a stress of 50 MPa are relevant. That is, from that moment on, it is considered that the material has reached its limit of structural resistance, and consequently, the collapse of the structure occurs. Additionally, in the figure, there are also small grey areas that indicate that in these areas, the maximum stress (50 MPa) has been exceeded, which corroborates that this instant is considered the moment in which the collapse of the structure.

Table 8 shows the comparison between the effective failure stress values of each model and those obtained by the finite element analysis. With these data, it can be verified that there is a coherence and high correlation between the experimental tests and the finite element models. This will allow us to make more complex models, taking the failure criterion as the moment at which a stress of 50 MPa is reached.

## 4. Discussion

In the compression test of the analyzed models, it was observed that the force–displacement curves are linear with a single loading cycle and abrupt failure in all cases except in model D (auxetic 2), which presents two loading cycles and one more gradual breakage.

In view of the experimental tests carried out, it can be stated that design G (three-dimensional auxetic) is the model that supports the greatest breaking force and is also the most expensive to manufacture, as well as that design C (auxetic 1) is capable of withstanding the greatest deformation in the compression direction before breaking, having an average manufacturing cost in relation to the rest of the models. Additionally, design D (auxetic 2) has shown that in its breaking process, the fragments that fractured were not projected toward the outside of the piece, but rather an implosion phenomenon took place. The regions where a fracture occurred were not a starting point for the propagation of said fracture. However, in other designs such as A (rhombuses), B (hexagonal), E (Voronoi organic) or F (cubic with diagonals), a direction of fracture propagation is clearly seen, design D (auxetic 2) tends to show different decentralized fracture points.

Furthermore, model D (auxetic 2) maintains its integrity and stability despite suffering numerous partial fractures even when there is a continuous fracture surface that splits the model in two. In this case, the piece is divided into several parts and has zero resistance to traction efforts.

On the other hand, the H design pieces (organic with irregular mesh) show a very interesting behavior that can be highly useful in structures that need to ensure their integrity for safety reasons. This model is capable of resisting compression forces of more than 4000 N until it compresses approximately 5%. From this point on, a partial fracture allows the structure to continue compressing while resisting stresses of up to 2000 N, according to experimental results. In this way, the change in behavior warns of a partial rupture in a non-catastrophic way.

The finite element analysis of the models analyzed in the simulation of the compression test has made it possible to determine the breaking stresses and the failure mode of the different models. The high degree of similarity between the results of the experimental test and those obtained in the FEM analysis allows the simulation carried out with the considered hypotheses to be validated. In particular, the appropriateness of using the moment at which a stress of 50 MPa is reached as the model’s failure criterion has been confirmed.

Additionally, it has been observed that the initial failure of the models varies depending on the design. Similar behaviors are observed for each design, both in the model and in the experimental tests. Once the test failure occurs, a rapid progression of the breakage appears, while these dynamic effects have not been introduced in the model.

## 5. Conclusions

The following points provide the main conclusions of the presented research:

- Up to eight different models have been designed and manufactured, with two-dimensional and three-dimensional patterns of structures that could work as compression stress dissipative layers, with some being based on models found in the literature and others being bioinspired. The objective has been to achieve lightweight structures resistant to compressive stresses in critical service conditions where it is necessary to guarantee the safety of people and property.
- The manufacturing of the models has been carried out using SLA 3D printing technology. This system has been very useful in the creation of prototypes, allowing the manufacturing of complex geometries quickly and efficiently. It has also made it possible to evaluate and verify the functionality of the designed models.
- The experimental results of the compression tests have shown that the choice of one model or another will depend on the specific needs of each situation. Geometry is a determining factor in the behavior of structures. In situations where greater deformation is sought, a more flexible structure, such as model C (auxetic 1), will be required. On the other hand, given a greater need for stability and load, a more rigid and resistant structure, such as the G model (three-dimensional auxetic), will be preferable. Moreover, analyzing costs, the G model (three-dimensional auxetic) is the most expensive but also presents the best profitability since it has the highest force/cost ratio.
- On the other hand, it has been experimentally proven that the types of breakage are also different depending on the configuration they present. An assessment of the manufacturing cost of each of the models has also been made, considering all the factors that affect them. In this way, the relationship between strength at breakage and manufacturing cost is analyzed, which can be very relevant when deciding on one model or another for a given application.
- Furthermore, through finite element analysis, the behavior of the designed structures has also been evaluated, allowing their behavior and stress distribution to be predicted, which are key characteristics for the optimization of designs. The results obtained have allowed us to validate the simulation carried out with the considered hypotheses. With this, the ability to create and analyze more complex models in less time and optimize the selection of the most appropriate alternative has been achieved.

## Figures and Tables

**Table 1:** SLA 3D printing parameters.

Layer Height	0.05 mm
Bottom Layer Count	8
Transition Type	Linear
Exposure Time	6 s
Bottom Exposure Time	50 s
Lifting Distance	5 mm
Lifting Speed	65 mm/min
Retract Speed	150 mm/min

**Table 2:** Resin technical properties.

Hardness/Contraction	84D/7.1%
Viscosity (25 °C)	150–200 mPa.s
Density (liquid)	1.100 g/cm^3^
Density (solid)	1.195 g/cm^3^
Bending resistance	59–70 MPa
Compressive strength	36–53 MPa
Elongation at break	14.2
Price	36.99 EUR/kg

**Table 3:** Characteristics of the eight manufactured structure models.

Pattern Type	Model	Lattice Dimensions (mm)	Truss Diameter (mm)	Mass (g)	Density (g/cm^3^)
2D	(A) Rhombuses	12.5 × 12.5	1.6	93	0.372
	(B) Hexagonal	10.2 × 9.1	1.7	93	0.372
	(C) Auxetic 1	19.5 × 13.9	1.4	94	0.376
	(D) Auxetic 2	18.9 × 12.1	0.9	95	0.380
	(E) Organic Voronoi	Irregular	Irregular	102	0.408
3D	(F) Cubic with diagonals	12.4 × 12.4	2.7	92	0.368
	(G) 3D auxetic	17.7 × 9.2	3.2	94	0.376
	(H) Organic–irregular mesh	Irregular	Irregular	93	0.372

**Table 4:** Manufacturing cost of the eight models printed by SLA.

Pattern Type	Model	Mass (g)	Printing Time (h)	Material Cost (EUR)	Labor Cost (EUR)	Operating Cost (EUR)	Manufacturing Cost (EUR)
2D	(A) Rhombuses	93	10.2	3.44	54.34	0.36	58.14
	(B) Hexagonal	93	9.82	3.44	52.37	0.34	56.15
	(C) Auxetic 1	94	9.82	3.48	52.39	0.34	56.21
	(D) Auxetic 2	95	10.3	3.51	54.9	0.36	58.77
	(E) Organic Voronoi	102	10.53	3.77	56.19	0.37	60.33
3D	(F) Cubic with diagonals	92	10.15	3.40	54.08	0.35	57.83
	(G) 3D auxetic	94	15.32	3.48	80.97	0.54	84.99
	(H) Organic with irregular mesh	93	14.8	3.44	78.25	0.52	82.21

**Table 5:** Experimental outputs of 8 proposed models.

Patterns	Models	Mean Breaking Force (kN)	Mean Break Deformation (%)	Mean Effective Breaking Stress (MPa)	Mean Young’s Modulus (MPa)	Absolute Difference (kN)	Relative Difference (%)
2D	(A) Rhombuses	2.80	3.81	1.12	796.01	1.45	107
	(B) Hexagonal	4.58	3.87	1.83	1279.43	3.23	239
	(C) Auxetic 1	1.49	4.83	0.60	401.95	0.14	10
	(D) Auxetic 2	1.35	3.20	0.54	221	-	-
	(E) Organic Voronoi	4.95	3.18	1.98	1588.47	3.6	267
3D	(F) Cubic and diagonals	5.77	4.05	2.31	1667	4.42	327
	(G) 3D auxetic	13.39	4.02	5.36	3389.3	12.04	892
	(H) Organic and irreg. mesh	4.34	5.01	1.74	1410.63	2.99	221

**Table 6:** Comparative analysis between breaking force and manufacturing costs.

Patterns	Models	Mean Breaking Force (kN)	Manufacturing Cost (EUR)	Force/Cost Ratio (kN/EUR)	Absolute Difference (kN/EUR)	Relative Difference (%)
2D	(A) Rhombuses	2.80	58.14	0.05	0.03	150
	(B) Hexagonal	4.58	56.15	0.08	0.06	300
	(C) Auxetic 1	1.49	56.21	0.03	0.01	50
	(D) Auxetic 2	1.35	58.77	0.02	-	-
	(E) Organic Voronoi	4.95	60.33	0.08	0.06	300
3D	(F) Cubic and diagonals	5.77	57.83	0.1	0.08	400
	(G) 3D auxetic	13.39	84.99	0.16	0.14	700
	(H) Organic and irreg. mesh	4.34	82.21	0.05	0.03	150

**Table 7:** Type of breakage of each model.

Patterns	Models	Type of Breakage
2D	(A) Rhombuses	Ductile
	(B) Hexagonal	Ductile
	(C) Auxetic 1	Fragile
	(D) Auxetic 2	Fragile
	(E) Organic Voronoi	Fragile
3D	(F) Cubic and diagonals	Fragile
	(G) 3D auxetic	Other
	(H) Organic and irreg. mesh	Other

**Table 8:** Comparison of results between experimental tests and finite element model (FEM) outputs.

Pattern	Models	Effective Breaking Stress (MPa)	Breaking Stress FEM (MPa)	Absolut Value (MPa)	Relative Value (%)
2D	(A) Rhombuses	1.12	1.06	0.06	5.36
	(B) Hexagonal	1.83	1.68	0.15	8.19
	(C) Auxetic 1	0.60	0.65	0.05	8.33
	(D) Auxetic 2	0.54	0.6	0.06	11.11
	(E) Organic Voronoi	1.98	1.89	0.09	4.54
3D	(F) Cubic and diagonals	2.31	2.21	0.1	4.33
	(G) 3D auxetic	5.36	4.84	0.52	9.70

## Data Availability

Dataset is available upon request from the authors.
